# The impact of early‐stage COVID‐19 pandemic on the diagnosis and treatment of gastric cancer: A cross‐sectional study using a large‐scale cancer registry in Hiroshima, Japan

**DOI:** 10.1002/deo2.180

**Published:** 2022-11-06

**Authors:** Masanobu Kodama, Daisuke Miyamori, Keishi Kanno, Masanori Ito

**Affiliations:** ^1^ Graduate School of Public Health Hiroshima University Hiroshima Japan; ^2^ Department of General Internal Medicine Hiroshima University Hospital Hiroshima Japan

**Keywords:** behavioral change, cancer screening, COVID‐19, Esophagogastroduodenoscopy, gastric cancer

## Abstract

**Background:**

After the confirmation of coronavirus infection in Japan, a behavioral change caused people and physicians to refrain from visiting hospitals or undergoing examinations. This study aimed to assess how the trend of diagnosis in gastric cancers changed, and how it affected the therapeutic strategies and the interval from diagnosis to treatment during the COVID‐19 pandemic.

**Methods:**

We use 15 cancer‐designated hospitals’ registries in Hiroshima, Japan. The target period was March to December 2020, and the same period in 2019 was set as the control period. The monthly mean of diagnoses and the interval from diagnosis to treatment were compared overall and separately by age, treatment procedure, diagnostic process, and clinical stage.

**Result:**

In 2020, the monthly mean (standard deviation [SD]) of patients was 192.2 (29.9), a significant 20.1% decrease from 240.7 (20.7) in 2019 due to older age and curative treatment groups. By reason for performing endoscopy, the change rate in cancer screening, endoscopic follow‐up, and symptomatic status were ‐27.0%, ‐18.0%, and ‐17.3%, respectively. Meanwhile, the interval (days) from diagnosis to treatment (SD) was 37.8 (26.5) in 2020, significantly shorter than 46 (31.5) in 2019.

**Conclusion:**

From 2019 to 2020, we observed a significant decrease in the diagnosis of curable early‐stage gastric cancer and treatments, although the interval from diagnosis to treatment decreased. This study suggests that cancer screening played a significant role in the decline in cancer diagnosis that occurred during the COVID‐19 pandemic. Even under COVID‐19 pandemic conditions, there should be an awareness of cancer screening and endoscopic follow‐up.

## INTRODUCTION

The first case of coronavirus disease 2019 (COVID‐19) was identified in Wuhan, China in December 2019.^1^ The disease has subsequently spread worldwide, led to an ongoing pandemic, and has been a global health concern.^2^, ^3^ The COVID‐19 pandemic has strained health system capacity worldwide, reduced access to health care for patients, and affected the diagnosis and treatment of diseases.^4^

Gastric cancer (GC) is a major global health threat, and 1.22 million incident cases occurred worldwide where nearly 865,000 people died in 2019, whereas 126,000 cases were diagnosed in Japan.^5^ Of those, diagnosis rates by cancer screening, endoscopic follow‐up, and symptomatic patients were 23.4%, 34.8%, and 39.8%, respectively.^6^ Since GC is mostly caused by *Helicobacter pylori*, the number of patients is expected not to change drastically.^7^, ^8^

In the pandemic era, cancer screening by upper gastrointestinal endoscopy was extremely suppressed by 50%–85% to avoid transmission of COVID‐19 infection, which may cause delays in cancer detection and initiation of treatment.^9^, ^10^ During the COVID‐19 pandemic period, a delay in the detection and treatment of GC had a substantial impact on mortality in the future. Therefore, assessing the diagnostic process for reduced GC diagnosis is necessary to avoid preventable cancer death.

The pandemic in Japan has resulted in fewer outpatient visits, which may have extended the post‐diagnosis‐to‐treatment process.^11^ Past studies have reported prolonged time from diagnosis to initial treatment in breast cancer,^12^ however, no study examined the interval from diagnosis to initial treatment during the pandemic in GC.

This study aimed to assess how the early stage of the COVID‐19 pandemic had an impact on the detection and treatment of GC. The present study investigates the difference in the number of patients with GC between 2019, a period before the pandemic, and 2020, a period since the pandemic began, using a hospital‐based cancer registry from 15 designated cancer hospitals. These results might be useful to make a countermeasure against the impact of the COVID‐19 pandemic on national and regional cancer control.

## METHODS

### Study design and setting

This study is a multicenter, cross‐sectional retrospective study.

### Hospital‐based cancer registry in Hiroshima

In Hiroshima, a hospital‐based cancer registry has been implemented mainly in designated cancer hospitals.^13^, ^14^ It includes diagnostic and therapeutic information about all newly diagnosed cancer at a medical institution. A designated cancer hospital provides highly qualified cancer care with nationwide equalization.

We extracted the information from all the institutions located in Hiroshima, consisting of one university hospital, one cancer specialized center, and 13 tertiary hospitals.

### Exposure and control period

The COVID‐19 period was defined as between March and December 2020, because a global pandemic of COVID‐19 has been declared by the World Health Organization on March 11, 2020.^15^ The control period was defined as between March and December 2019. We compared the monthly mean and interval from diagnosis to treatment among GC patients in the COVID‐19 period to those in the control period. Moreover, we used publicly available open data on COVID‐19 surges in the Hiroshima Prefecture.

### Eligibility criteria

Patients who were newly diagnosed as GC in the COVID‐19 period or control period were included in the study. All enrolled individuals are aggregated in an anonymized state.

### Data extraction

We gathered data regarding age, gender, pathological finding, clinical stage (CS) of GC before treatment, diagnostic process, treatment category, and date of diagnosis and treatment initiation. In the cancer registry, the diagnostic process was categorized as cancer screening, endoscopic follow‐up, symptomatic patients, and others. Cancer screening was defined as a diagnosis of GC after cancer screening by municipals, obligated health checkups by companies, or self‐funded health screening. An endoscopic follow‐up is a periodic follow‐up for patients who were previously diagnosed with upper gastrointestinal diseases, such as Barrett's esophagus and gastritis, or who were diagnosed with GC in the past and had surgery or endoscopic treatment. The CS of GC was based on the Union for International Cancer Control staging system, eighth edition.^16^

### Definition of GC and treatment

GC was defined in this study as code C16 by ICD‐O3.^17^ That is, GC in this study included carcinoma, carcinoid tumor, endocrine cell carcinoma, gastrointestinal stromal tumor, sarcoma, leiomyosarcoma, and lymphoma. Treatment categories consisted of endoscopic treatment, laparoscopic surgery, open surgery, chemotherapy, radiation therapy, endocrine therapy, other therapy, and no treatment. If patients received more than one treatment, we defined initial treatment as the treatment evaluated in this study.

### Assessment of the monthly mean

The outcome was the monthly mean of GC patients among each subgroup category of CS, diagnostic process, and treatment. The outcome was based on the date of GC diagnosis and compared between the COVID‐19 and the control period. We also conducted a subgroup analysis of the diagnostic process in each treatment and CS category. The change rate was defined as the percentage change in monthly mean during the COVID‐19 period relative to the control period.

### Assessment of interval from diagnosis to treatment

We evaluated the interval from the date of diagnosis to initial treatment among each category of CS, age, sex, diagnostic process, and treatment. The date of initial diagnosis was defined if the patients were diagnosed with pathological findings.

### Statistical analysis

Patient characteristics were expressed as means with standard deviation for continuous variables, and as numbers with percentages for categorical variables. Differences in the means of continuous variables were compared using an unpaired student t‐test. Categorical variables were compared using the chi‐square test. Differences were considered statistically significant at *P* <0.05. Statistical analyses were performed with R version 4.02 (The R Foundation for Statistical Computing, Vienna, Austria).

### Ethical considerations

This study was approved by the Ethical Committee for Epidemiology of Hiroshima University (E‐2660). This study was conducted by the Declaration of Helsinki. The requirement for informed consent was waived as the data was gathered and analyzed anonymously.

## RESULTS

### Baseline characteristics

This study evaluated 4329 patients including 2407 patients from before the COVID‐19 pandemic and 1922 patients during the COVID‐19 pandemic. The baseline characteristics of study 1 are shown in Table 1. The mean age (standard deviation [SD]) of GC patients in the COVID‐19 period and control group was 73.6 (10.7) and 73.3 (10.8) years old, respectively. Regarding age, gender, CS, pathology, the diagnostic process, and initial treatment, a significant difference was not observed between COVID‐19 and the control period. As the population in the local community was 2,808,000 in 2019 and 2,795,000 in 2020,^18^ the prevalence of newly diagnosed GC was significantly lower in 2020 than in 2019 (85.7 vs. 68.5 per 100,000 population; *p* < 0.001), respectively.

**TABLE 1:** Patient characteristics

	**Total**	**Control period**	**COVID‐19 period**	
	***n* = 4329**	**(*n* = 2407)**	**(*n* = 1922)**	***p*‐value**
Age (years)				0.46
Mean (SD)	73.5 (10.8)	73.6 (10.7)	73.3 (10.8)	
Gender				0.19
Male, *n* (%)	2948 (68.1%)	1619 (67.3%)	1329 (69.1%)	
Process of detection, *n* (%)				0.35
Screening	1007 (23.3%)	582 (24.2%)	425 (22.1%)	
Endscopic follow‐up	1665 (38.5%)	915 (38.0%)	750 (39.0%)	
Symptomatic	1599 (36.9%)	875 (36.4%)	724 (37.7%)	
Others	58 (1.3%)	35 (1.5%)	23 (1.2%)	
Clinical stage, *n* (%)				0.12
Stage 0	1 (0.0%)	1 (0.0%)	0 (0.0%)	
Stage 1	2746 (63.4%)	1559 (64.8%)	1187 (61.8%)	
Stage 2	371 (8.6%)	214 (8.9%)	157 (8.2%)	
Stage 3	330 (7.6%)	176 (7.3%)	154 (8.0%)	
Stage 4	616 (14.2%)	314 (13.0%)	302 (15.7%)	
Unknown	265 (6.1%)	143 (5.9%)	122 (6.3%)	
Pathology, *n* (%)				0.28
pap	115 (2.7%)	65 (2.7%)	50 (2.6%)	
tub	2604 (60.2%)	1475 (61.3%)	1129 (58.7%)	
por	822 (19.0%)	436 (18.1%)	386 (20.1%)	
sig	46 (1.1%)	25 (1.0%)	21 (1.1%)	
muc	211 (4.9%)	117 (4.9%)	94 (4.9%)	
Lymphoma	140 (3.2%)	73 (3.0%)	67 (3.5%)	
NET	40 (0.9%)	24 (1.0%)	16 (0.8%)	
GIST	157 (3.6%)	88 (3.7%)	69 (3.6%)	
Un_differen	111 (2.6%)	66 (2.7%)	45 (2.3%)	
Other carcinomas	24 (0.6%)	15 (0.6%)	9 (0.5%)	
Unknown	59 (1.4%)	23 (1.0%)	36 (1.9%)	
Initial Treatment, *n* (%)				0.14
Endoscopy	1811 (41.8%)	1005 (41.8%)	806 (41.9%)	
Laparoscopy	687 (15.9%)	402 (16.7%)	285 (14.8%)	
Open	718 (16.6%)	403 (16.7%)	315 (16.4%)	
Chemo	430 (9.9%)	223 (9.3%)	207 (10.8%)	
Radiation	25 (0.6%)	9 (0.4%)	16 (0.8%)	
Palliative care	323 (7.5%)	173 (7.2%)	150 (7.8%)	
Unknown	335 (7.7%)	192 (8.0%)	143 (7.4%)	

Abbreviations: GIST, gastrointestinal stromal tumor; muc, mucinous adenocarcinoma; NET, neuroendocrine tumor; pap, papillary adenocarcinoma; por, poorly differentiated adenocarcinoma; SD, standard deviation; sig, signet‐ring cell carcinoma; tub, tubular adenocarcinoma; un_differen, undifferentiated adenocarcinoma.

Other carcinomas include adenosquamous carcinoma, carcinoma with lymphoid stroma, hepatic cell carcinoma, squamous cell carcinoma, hepatoid carcinoma, and medually carcinoma.

Chi‐square test; unpaired t‐test; and wilcoxon ranksum test.

### Number of patients diagnosed with GC and COVID‐19 in the target and comparison periods

**FIGURE 1:**
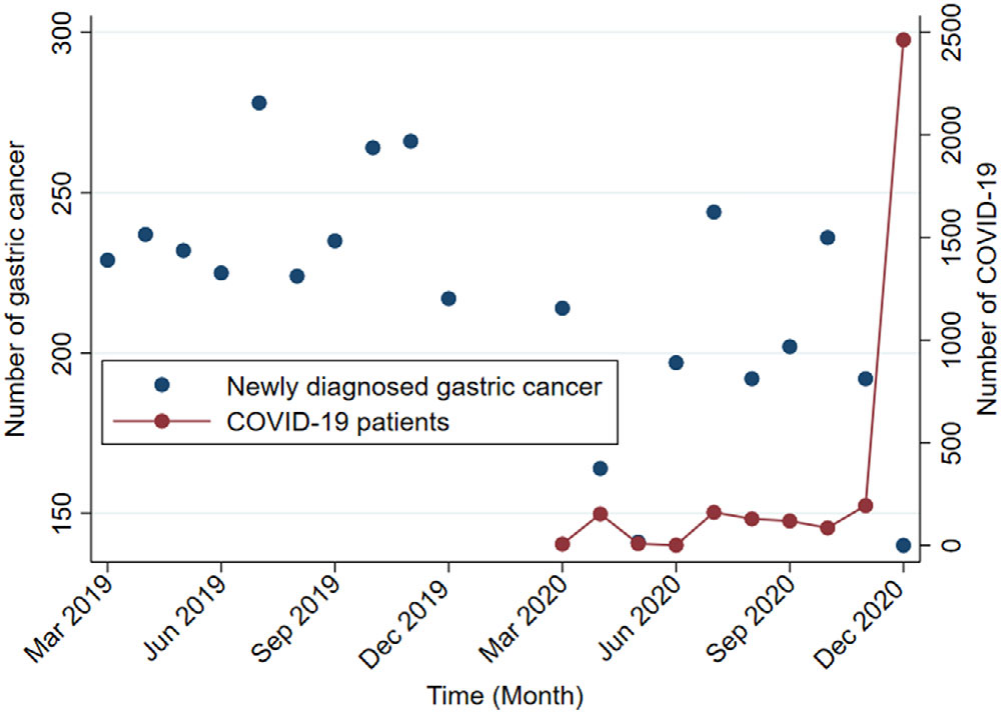
Number of diagnosed gastric cancer and COVID‐19 patients in target and comparison periods. The left axis indicates the number of gastric cancer patients each month, while the right axis indicates the number of COVID‐19 in Hiroshima Prefecture. Newly diagnosed gastric cancer was plotted monthly in blue during the control period (from Mar to Dec 2019) and target period (from Mar to Dec 2020), while COVID‐19 was plotted in red during the target period.

Figure 1 plots the number of GC diagnoses and corona patients in each period; COVID‐19 surges were observed in April, July, and December, with a declining trend in the number of GC in the same or the following month.

### Change in the monthly mean of GC patients in each CS, the diagnostic process, pathological findings, and initial treatment category

Table 2 shows the monthly mean of GC patients among categories of age, CS, pathology, initial treatment, and diagnostic process. The monthly mean (SD) were significantly reduced from 240.7 (20.6) patients per/month to 192.2 (29.9) per/month (*p* = 0.001). Regarding categories of age, aged over 60 were significantly decreased, whereas the category aged less than 60 were not decreased significantly. Among categories of diagnostic process, monthly mean (SD) were significantly reduced in all the category of cancer screening (58.2 [13.4] vs. 42.5 [13.9]; *p* = 0.02), endoscopic follow‐up (91.5 [7.9] vs. 75.0 [14.1]; *p* = 0.005) and presence of symptoms (87.5 [8.8] vs. 72.4 [16.8]; *p* = 0.02). Significant decreases in the monthly mean (SD) of GC patients were also observed in the clinical category of CS1 (155.9 [16.5] vs. 118.7 [25.4]; *p* = 0.003), and CS2 (21.4 [5.8] vs. 15.7 [4.2]; *p* = 0.02), whereas CS3 and CS4 were not significantly different. In the category of treatment options, significant decrease was shown in endoscopic treatment (100.5 [10.6] vs. 80.6 [20.1]; *p* = 0.01), laparoscopic surgery (40.2 [7.0] vs. 28.5 [8.3]; *p* = 0.003) and open surgery (40.3 [10.1] vs. 31.5 [7.1]; *p* = 0.04), which were potentially resectable treatment procedures. However, no differences were observed in the chemotherapy group, other treatment groups (radiation and endocrine therapy), and non‐treatment groups.

**TABLE 2:** Comparison of Patients frequency among Primary outcome group

	**Cumulative no**.		**Per month, mean (SD)**			
	**Control**	**COVID‐19**	**Control**	**COVID‐19**	**Change rate (%)**	***p*‐value**
Total	2407	1922	240.7 (20.6)	192.2 (29.9)	−20.1	0.001
Age category (years)						
20‐29	5	4	0.5 (0.85)	0.4 (0.699)	−20.0	0.777
30‐39	13	12	1.3 (1.636)	1.2 (1.033)	−7.7	0.872
40‐49	66	57	6.6 (1.955)	5.7 (2.751)	−13.6	0.41
50‐59	130	133	13 (3.091)	13.3 (4.923)	2.3	0.872
60‐69	480	367	48 (7.211)	36.7 (9.81)	−23.5	0.009
70‐79	994	803	99.4 (10.002)	80.3 (14.182)	−19.2	0.003
80‐89	613	471	61.3 (8.757)	47.1 (11.958)	−23.2	0.007
90‐99	103	73	10.3 (2.003)	7.3 (4.001)	−29.1	0.048
100 or more	3	2	0.3 (0.483)	0.2 (0.422)	−33.3	0.628
Process of detection						
Screening	582	425	58.2 (13.4)	42.5 (13.9)	−27.0	0.02
Endscopic follow‐up	915	750	91.5 (7.9)	75 (14.1)	−18.0	0.005
Presence of symptom	875	724	87.5 (8.8)	72.4 (16.8)	−17.3	0.02
Others	35	23	3.5 (2.1)	2.3 (1.8)	−34.3	0.19
Stage						
Stage 0	1	0	0.1 (0.32)	0 (0)	−100.0	0.33
Stage 1	1559	1187	155.9 (16.5)	118.7 (25.4)	−23.9	0.003
Stage 2	214	157	21.4 (5.8)	15.7 (4.2)	−26.6	0.02
Stage 3	176	154	17.6 (5.8)	15.4 (4.6)	−12.5	0.56
Stage 4	314	302	31.4 (5.1)	30.2 (5.9)	−3.8	0.84
Unknown	143	122	14.3 (2.9)	12.2 (3)	−14.7	0.02
Initial treatment						
Endoscopy	1005	806	100.5 (10.6)	80.6 (20.1)	−19.8	0.01
laparoscopy	402	285	40.2 (7)	28.5 (8.3)	−29.1	0.003
Open surgery	403	315	40.3 (10.1)	31.5 (7.1)	−21.8	0.04
Chemotherapy	223	207	22.3 (3.1)	20.7 (3.2)	−7.2	0.27
Radiation therapy	9	16	0.9 (1.1)	1.6 (1)	77.8	0.15
Other therapies	0	0	0 (0)	0 (0)	0.0	NA
Palliative care	173	150	17.3 (3.5)	15 (3.7)	−13.3	0.17
Unknown	192	143	19.2 (4.8)	14.3 (5)	−25.5	0.04

Abbreviations: COVID‐19, coronavirus disease 2019; NA, not applicable; SD, standard deviation.

### Changes in mean days since the diagnosis to initiate treatment of GC patients by diagnostic process, CS, and treatment categories

Table 3 shows the interval post‐diagnosis to treatment in categories of the detection process, CSs, and treatment procedures. A significant decrease in days (SD) from diagnosis to treatment was observed in the COVID‐19 period compared to that in the control period (46 [31.5] vs. 37.8 [26.5]; *p* < 0.001). Among subgroup of detection process, interval days (SD) from diagnosis to treatment were significantly reduced in all the category of cancer screening (54.4 [29.5] vs. 42.7 [25.7]; *p* < 0.001), endoscopic follow‐up (46.1 [32.5] vs. 39.5 [29.3]; *p* < 0.001) and presence of symptoms (38.7 [26.7] vs. 32.6 [22.7]; *p* < 0.001). Significant decrease in the monthly mean (SD) of GC patients were also observed in all the clinical category of CS1 (53.4 [31.8] vs. 44 [27.7]; *p* = 0.02), CS2 (38.3 [24.2] vs. 32.4 [16.6]; *p* = 0.02), CS3 (33.4 [17.3] vs. 28.7 [17.2]; *p* = 0.02), and CS4 (27 [21.2] vs. 22.8 [15.8]; *p* = 0.02). In the category of treatment options, significant decrease was shown in endoscopic treatment (50 [32.3] vs. 40.6 [28.2]; *p* < 0.001), and laparoscopic surgery (54 [29.4] vs. 40.6 [28.2]; *p* < 0.001). However, no differences were observed in open surgery, chemotherapy, and radiation treatment groups.

**TABLE 3:** Intervals between first diagnosis and initial treatment

	**Number of patients**		**Mean (SD)**		
	**Control**	**COVID‐19**	**Control**	**COVID‐19**	***p*‐value**
All	2042	1629	46 (31.5)	37.8 (26.5)	<0.001
Female	659	491	44.1 (35.6)	29.4 (26.3)	<0.001
Male	1383	1138	46.9 (38.8)	32.4 (26.5)	<0.001
Age category (y)					
20‐29	4	4	47.8 (40.8)	35.3 (24.8)	0.76
30‐39	13	10	43.3 (32.4)	25.9 (14.9)	0.25
40‐49	61	53	46.6 (39.8)	27.7 (27.9)	0.20
50‐59	119	119	53 (43.5)	30.2 (33.1)	0.02
60‐69	448	330	46.2 (36.6)	28.6 (23.4)	<0.001
70‐79	875	725	45.7 (37.9)	33.9 (26.4)	<0.001
80‐89	479	363	44.6 (37.1)	30.3 (26.7)	<0.001
90 or over	43	25	47 (34.2)	32.4 (31.1)	0.12
Process of detection					
Screening	555	402	54.4 (29.5)	42.7 (25.7)	<0.001
Endscopic follow‐up	775	647	46.1 (32.5)	39.5 (29.3)	<0.001
Symptomatic	698	575	38.7 (26.7)	32.6 (22.7)	<0.001
Others	14	5	79 (101)	31.8 (18.3)	0.32
Stage					
Stage 0	1	0	()	()	0.33
Stage 1	1396	1072	53.4 (44)	31.8 (27.7)	<0.001
Stage 2	183	131	38.3 (32.4)	24.2 (16.6)	0.02
Stage 3	155	146	33.4 (28.7)	17.3 (17.2)	0.02
Stage 4	225	202	27 (22.8)	21.2 (15.8)	0.02
Unknown	82	78	12.7 (18.6)	28.5 (28.4)	0.19
Treatment					
Endoscopy	1005	806	50 (40.6)	32.3 (28.2)	<0.001
Laparoscopy	402	285	53.6 (43.3)	29.5 (24.9)	<0.001
Open surgery	403	315	37.2 (34.4)	23.7 (24.7)	0.13
Chemotherapy	223	207	28.3 (25.3)	21.5 (18.4)	0.12
Radiation therapy	9	16	102.7 (32.7)	127.8 (32.9)	0.05

Abbreviations: COVID‐19, coronavirus disease 2019; SD, standard deviation.

### Changes in the monthly mean of GC patients among CSs and treatment categories by the diagnostic process

Table 4 shows the difference in the monthly mean in each diagnostic process among CS categories. The difference in the monthly mean in each diagnostic process was evaluated at each CS in Table 4. In the category of CS1, significant decreases were shown in the screening group (50.4 [10.4] vs. 36.3 [12.2]; *p* = 0.01), endoscopic follow‐up group (68.2 [4.2] vs. 53.6 [11.9]; *p* = 0.002) and symptomatic group (36.0 [7.1] vs. 28.4 [8.0]; *p* = 0.04), whereas processes of detection in other CSs were not significantly decreased.

**TABLE 4:** Comparison of the numbers of gastric cancer and the process of detection before and during the COVID‐19 pandemic for clinical stage subgroup

	**Cumulative No**.		**Per month, mean (SD)**			
**Treatment category**	**Control**	**COVID‐19**	**Control**	**COVID‐19**	**Change rate (%)**	***p*‐value**
Stage 0						
Total	1	0	0.1 (0.31)	0 (0)	−100.0	0.33
Process of detection						
Symptomatic	1	0	0.1 (0.31)	0 (0)	−100.0	0.33
Stage 1						
Total	1559	1187	155.9 (16.5)	118.7 (25.4)	−23.9	0.003
Process of detection						
Screening	504	363	50.4 (10.4)	36.3 (12.2)	−28.0	0.01
Endscopic follow‐up	682	536	68.2 (4.2)	53.6 (11.9)	−21.4	0.002
Symptomatic	360	284	36 (7.1)	28.4 (8)	−21.1	0.04
Others	13	4	1.3 (1.4)	0.4 (0.5)	−69.2	0.08
Stage 2						
Total	215	157	21.5 (5.8)	15.7 (4.2)	−27.0	0.02
Process of detection						
Screening	30	23	3 (2.4)	2.3 (2.2)	−23.3	0.5
Endscopic follow‐up	70	48	7 (2.6)	4.8 (2.3)	−31.4	0.06
Symptomatic	114	86	11.4 (4.1)	8.6 (3.9)	−24.6	0.12
Stage 3						
Total	176	154	17.6 (5.8)	15.4 (4.6)	−12.5	0.56
Process of detection						
Screening	15	13	1.5 (1.1)	1.3 (1.1)	−13.3	0.68
Endscopic follow‐up	41	41	4.1 (3.1)	4.1 (1.9)	0.0	1
Symptomatic	117	99	11.7 (4.1)	9.9 (3.3)	−15.4	0.29
Others	3	1	0.3 (0.5)	0.1 (0.3)	−66.7	0.29
Stage 4						
Total	314	302	31.4 (5.1)	30.2 (5.9)	−3.8	0.84
Process of detection						
Screening	18	17	1.8 (0.9)	1.7 (1.9)	−5.6	0.77
Endscopic follow‐up	46	59	4.6 (2)	5.9 (2)	28.3	0.16
Symptomatic	247	226	24.7 (4)	22.6 (5.8)	−8.5	0.36
Others	3	0	0.3 (1.4)	0 (1.5)	–100.0	0.07
Unknown						
Total	143	122	14.3 (2.9)	12.2 (3)	−14.7	0.02
Process of detection						
Screening	15	9	1.5 (1.5)	0.9 (1)	−40.0	0.18
Endscopic follow‐up	76	66	7.6 (1.6)	6.6 (1.4)	−13.2	0.12
Symptomatic	36	29	3.6 (1.3)	2.9 (1.4)	−19.4	0.09
Others	16	18	1.6 (1.4)	1.8 (1.5)	12.5	0.76

Abbreviations: COVID‐19, coronavirus disease 2019; SD, standard deviation.

### Changes in the monthly mean of GC patients among treatment categories by the diagnostic process

Table 5 shows the difference in the monthly mean of patients in each diagnostic process among treatment categories. In the endoscopic resection group and laparoscopic rection group, significant decreases in the monthly mean (SD) were observed in the screening groups and endoscopic follow‐up groups. In the endoscopic treatment category, the monthly mean was significantly decreased in screening (33.9 [6.0] vs. 25.6 [9.5]; *p* = 0.03) and endoscopic follow‐up (48.6 [3.9] vs. 41.0 [8.6]; *p* = 0.02) group, respectively. In addition, in the laparoscopic treatment group, the monthly mean was significantly decreased in screening (13.3 [4.3] vs. 8.2 [2.8]; *p* = 0.008) and endoscopic follow‐up (13.0 [2.6] vs. 8.6 [4.9]; *p* = 0.009) group, respectively. In the open surgery treatment category, none of the subgroups in detection were significant.

**TABLE 5:** Comparison of the numbers of gastric cancer and the process of detection before and during the COVID‐19 pandemic for treatment subgroup

	**Cumulative no**.		**Per month, mean (SD)**			
**Treatment category**	**Control**	**COVID‐19**	**Control**	**COVID‐19**	**Change rate (%)**	***p*‐value**
Endoscopic resection						
Total	1005	806	100.5 (12.2)	80.6 (18.3)	−19.8	0.01
Process of detection						
Screening	339	256	33.9 (6)	25.6 (9.5)	−24.5	0.03
Endscopic follow‐up	486	410	48.6 (3.9)	41 (8.6)	−15.6	0.02
Symptomatic	172	136	17.2 (6.5)	13.6 (4.9)	−20.9	0.18
Others	8	4	0.8 (1)	0.4 (0.7)	−50.0	0.32
Laparoscopic resection						
Total	402	285	40.2 (5.7)	28.5 (7.8)	−29.1	0.002
Process of detection						
Screening	133	82	13.3 (4.3)	8.2 (2.8)	–38.3	0.008
Endscopic follow‐up	130	86	13 (2.6)	8.6 (4.9)	−33.8	0.009
Symptomatic	137	116	13.7 (4.3)	11.6 (4.6)	–15.3	0.25
Others	2	1	0.2 (0.4)	0.1 (0.3)	−50.0	0.56
Open surgery						
Total	403	315	40.3 (7.6)	31.5 (5)	−21.8	0.03
Process of detection						
Screening	56	33	5.6 (4)	3.3 (1.5)	−41.1	0.09
Endscopic follow‐up	120	98	12 (5.3)	9.8 (1.8)	−18.3	0.14
Symptomatic	227	184	22.7 (4.7)	18.4 (5.3)	−18.9	0.09
Others	0	0	0 (0)	0 (0)		NA
Chemotherapy						
Total	223	207	22.3 (3.3)	20.7 (5.5)	–7.2	0.37
Process of detection						
Screening	25	29	2.5 (1.9)	2.9 (2.4)	16.0	0.77
Endscopic follow‐up	38	47	3.8 (1.7)	4.7 (1.9)	23.7	0.08
Symptomatic	158	131	15.8 (3.2)	13.1 (4.2)	−17.1	0.06
Others	2	0	0.2 (0.4)	0 (0)	−100.0	0.15
Radiation therapy						
Total	9	16	0.9 (1.3)	1.6 (1.9)	77.8	0.28
Process of detection						
Screening	2	2	0.2 (0.7)	0.2 (0.4)	0.0	0.45
Endscopic follow‐up	1	6	0.1 (0.3)	0.6 (0.9)	500.0	0.07
Symptomatic	4	8	0.4 (0.7)	0.8 (1.1)	100.0	0.24
Others	2	0	0.2 (0.4)	0 (0)	−100.0	0.15
Palliative care						
Total	173	150	17.3 (3.6)	15 (3.7)	−13.3	0.2
Process of detection						
Screening	9	7	0.9 (1)	0.7 (0.8)	−22.2	0.63
Endscopic follow‐up	76	60	7.6 (3.1)	6 (2.3)	−21.1	0.23
Symptomatic	87	83	8.7 (3.1)	8.3 (2.1)	−4.6	0.73
Others	1	0	0.1 (0.3)	0 (0)	−100.0	0.33

Abbreviations: COVID‐19, coronavirus disease 2019; SD, standard deviation; NA, not applicable.

## DISCUSSION

We investigated the monthly mean of GC patients during the COVID‐19 period compared to the control period regarding diagnostic, clinical, and treatment categories using a hospital‐based cancer registry of 15 designated cancer hospitals in Hiroshima. The result of this study showed that the monthly mean was significantly decreased in the COVID‐19 period, compared to those in the control period, especially in the categories of diagnosed patients by cancer screening, older age, early CS, and potentially curative treatment procedures. On the other hand, the interval from diagnosis to treatment was significantly shortened rather than extended in the COVID‐19 period compared to the control period. These results indicate the need for novel cancer diagnostic strategies for crises such as COVID‐19 and emerging infectious diseases.

Previous studies have shown significant decreases in GC patients of older age, early CS, and curative treatment procedures.^10^, ^19^, ^20^, ^21^, ^22^, ^23^, ^24^ However, previous studies showed only in a small number of facilities or in foreign countries, therefore, we revealed that the trends were consistent in the tertiary medical region covering 2.9 million populations in Japan. In addition, the change rate of each diagnostic procedure has not been studied previously, for that reason, we analyzed and found that the monthly mean of GCs decreased especially in cancer screening. The possible mechanisms of decline in the number of GC diagnoses may be due to the following; 1) upper gastrointestinal endoscopy could be a risk of droplet infection, which discouraged less urgent health checkups and follow‐up examinations, and 2) COVID‐19 is associated with higher risk of mortality in the elderly, which may have caused them to refrain from receiving cancer screening and endoscopic follow‐up, or 3) to refrain from receiving detailed examination for abnormal findings in cancer screenings. However, the last one is unlikely, since the statistics of cancer screenings by municipalities in the Hiroshima Prefecture showed a decrease in total numbers (16,000 in 2019 vs. 12,700 in 2020) but not in the proportion of patients who underwent subsequent detailed examinations after an abnormal screening (73.9% in 2019 vs. 74.1% in 2020).^25^

In Japan, more than 3 million population‐based GC screenings were carried out in practice.^26^, ^27^ Because patients with early GC are unlikely to present with clinical symptoms, early detection through screening is essential for radical treatment.^28^ If GC were not treated, the progression time from early GC to advanced GC was 34–44 months.^29^ Once patients progress to advanced GC, the 5‐year survival rates of untreated GC were 46.2% in stage I and 0% in stages II–IV.^29^, ^30^ Since the 5‐year survival rates after endoscopic treatment and laparoscopic surgery both exceed 90%,^32^, ^33^ cancer screening should be actively promoted even in the COVID‐19 pandemic, and GC should be detected at an early stage when these treatment indications are effective, particularly in older people.

Meanwhile, this study was the first to show that even in the absence of a medical shortage, cancer screening was suppressed, which could lead to delayed diagnosis, while treatment was promptly provided. Past studies have shown that the COVID‐19 pandemic may cause a risk of cancer progression in non‐GC cancers by delaying the time from detection to treatment.^34^, ^35^, ^36^ In the present study, the interval was significantly shortened rather than extended in the COVID‐19 period, compared to the control period. This might be because, during the early stage of the COVID pandemic in Hiroshima, there was spare capacity in the floor occupancy rate in those cancer‐designated and non‐cancer‐designated general hospitals.^37^ Actually, of the 14,300 acute care beds in Hiroshima Prefecture, 400 beds were allocated for COVID‐19 as of 2020, which is only 2.8% of the total number of acute care beds.^38^, ^39^ The number of outpatients decreased^40^ and patients diagnosed with cancer could be consulted by specialists more quickly, thus shortening the interval from detection to treatment.

There are several strengths in this study. First, we used a large‐scale GC registry in Hiroshima and overcame the external validity of previous studies conducted in a small number of facilities. We evaluated 15 designated cancer hospitals in the same tertiary medical region, where regular cancer treatments can be completed within the region. Thus, all risk populations were included. Second, the results of this study can be attributed to the decrease in newly diagnosed GC patients and not to delays in treatment. As indicated above, this study did not find any delay in treatment intervention. Therefore, the results were not due to a lack of access to treatment or a shortage of medical care but were the result of a decrease in the frequency of upper gastrointestinal endoscopies.

Several limitations should be considered. First, we investigated GC patients who were newly diagnosed between March 2020 and December 2020, but not investigated the long‐term impact on GC outcomes, such as recurrence rate or survival rate. However, since the GC progress over several years and the impact of COVID‐19 on the diagnosis and treatment of GC were not consistent as shown in Figure 1, it is not plausible to estimate the long‐term outcome of patients in this study. Second, there might be other factors except for the COVID‐19 pandemic that affects the number of GC diagnosed and treated between COVID‐19 and control periods, such as the prevalence of *H. pylori* infection. Since it takes a certain period between *H. pylori* infection and the onset of the GC,^41^ the prevalence of GC is unlikely to decrease significantly in 2019–2020. Third, selection bias may exist since not all patients diagnosed with GC had been referred to cancer‐designated hospitals. However, it is unlikely that cancer‐designated hospitals rejected patients with GC, thus this bias would be non‐differential rather than differential.

In conclusion, the results of this study showed that the monthly mean of endoscopic treatments, laparoscopic surgery, and open surgery in the COVID‐19 period decreased significantly, compared to those in the control period. In contrast, for diagnosed cases, there was no delay in time to treatment. We also found that a decrease in the number of cases of GC was especially influenced by a decrease in the number of cancer screenings and endoscopic follow‐ups, especially in older people. These issues should be considered significant in developing novel strategies for cancer diagnosis and treatment of COVID‐19 and other emerging infectious diseases to avoid further unfortunate cases of delayed diagnosis and treatment initiation.

## CONFLICT OF INTEREST

The authors declare that they have no conflict of interest.
